# Constitutional mutation in *CDKN2A* is associated with long term survivorship in multiple myeloma: a case report

**DOI:** 10.1186/s12885-017-3715-5

**Published:** 2017-11-06

**Authors:** Vallari Shah, Kevin D. Boyd, Richard S. Houlston, Martin F. Kaiser

**Affiliations:** 1 Division of Molecular Pathology, The Institute of Cancer Research, London, UK; 2 Department of Haemato-Oncology, Royal Marsden Hospital, London, UK; 3 Division of Genetics and Epidemiology, The Institute of Cancer Research, London, UK

**Keywords:** Myeloma, Germline mutation, Survival, *CDKN2A*

## Abstract

**Background:**

Multiple Myeloma is a cancer of plasma cells associated with significantly reduced survival. Long term survivorship from myeloma is very rare and despite advances in its treatment the disease is generally considered incurable. We report a patient diagnosed with myeloma carrying a germline mutation of a tumour suppressor gene who has effectively been cured.

**Case presentation:**

A 36-year-old woman was diagnosed with IgG lambda myeloma in 1985. She was treated with melphalan chemotherapy followed by high-dose melphalan and autologous stem cell rescue and since remained in complete remission despite not having received any additional therapy. After eliciting a prior history of multiple primary melanomas and breast cancer, she was tested for and shown to be a carrier for a germline mutation in *CDKN2A*.

**Conclusions:**

This is the second case report of germline mutation of *CDKN2A* being associated with myeloma. *CDKN2A* is a stabiliser of p53. Long term survivorship after high dose DNA damaging chemotherapy with melphalan in this patient is compatible with an increased chemo-sensitivity due to impairment of the DNA repair pathway.

## Background

Multiple Myeloma (MM) is caused by the neoplastic proliferation of somatically mutated plasma cells and is associated with significant morbidity and mortality [1]. The use of alkylating agents such as melphalan to treat MM four decades ago led to the first appreciable improvement in patient outcome with survival rates of between 24 and 48 months after diagnosis [2]. The subsequent introduction of immunomodulatory agents, proteasome inhibitors and high-dose autologous stem cell transplantation, maintenance therapy, monoclonal antibodies and histone deacetylase inhibitors more recently has led to further improvements in patient outcome and median 5-year survival rates are typically now around 50% [3]. Despite these advances in treatment autologous stem cell transplantation has still been shown to be beneficial in extending survival [4].

There is however significant variation in outcome between patients with apparently same stage disease. Staging systems such as the international staging system (ISS) which uses serum albumin and β2-microglobulin concentrations and the Revised-ISS (R-ISS) incorporating some adverse genetic markers and lactate dehydrogenase at diagnosis attempt to predict patients’ outcome. These markers of adverse prognosis however cannot always accurately predict survival and there remain several factors that are currently unknown with regards to prognosis and response to treatment in myeloma. Of considerable interest is understanding why a very small number of patients have particularly long survivorship for what is essentially an incurable disease.

It is increasingly being recognised that, as well as the tumour profile, constitutional genotype also plays a role in determining patient outcome [5]. Here we report on an MM patient who has been in complete remission for over 30 years after only receiving first-line standard of care possibly being a consequence of also having hereditary Melanoma Syndrome.

## Case presentation

The patient, a 36-year-old woman, was diagnosed in 1985 with IgG lambda MM after presenting with tiredness and recurrent infections. She was found to be anaemic with a haemoglobin level of 73 g/l and thrombocytopenic with a platelet count of 85 × 10^9^/l. Further testing revealed a markedly raised paraprotein of 62 g/l with positive urinary Bence-Jones protein. There was evidence of immunosuppression with reduced levels of uninvolved IgA (0.1 g/l) and IgM (0.2 g/l) immunoglobulins. Her renal function was reduced as evidenced by a creatinine clearance of 57 ml/min. A skeletal survey revealed multiple lytic lesions in both her humeri and femora. A bone marrow biopsy confirmed a diagnosis of MM.

**Fig. 1:**
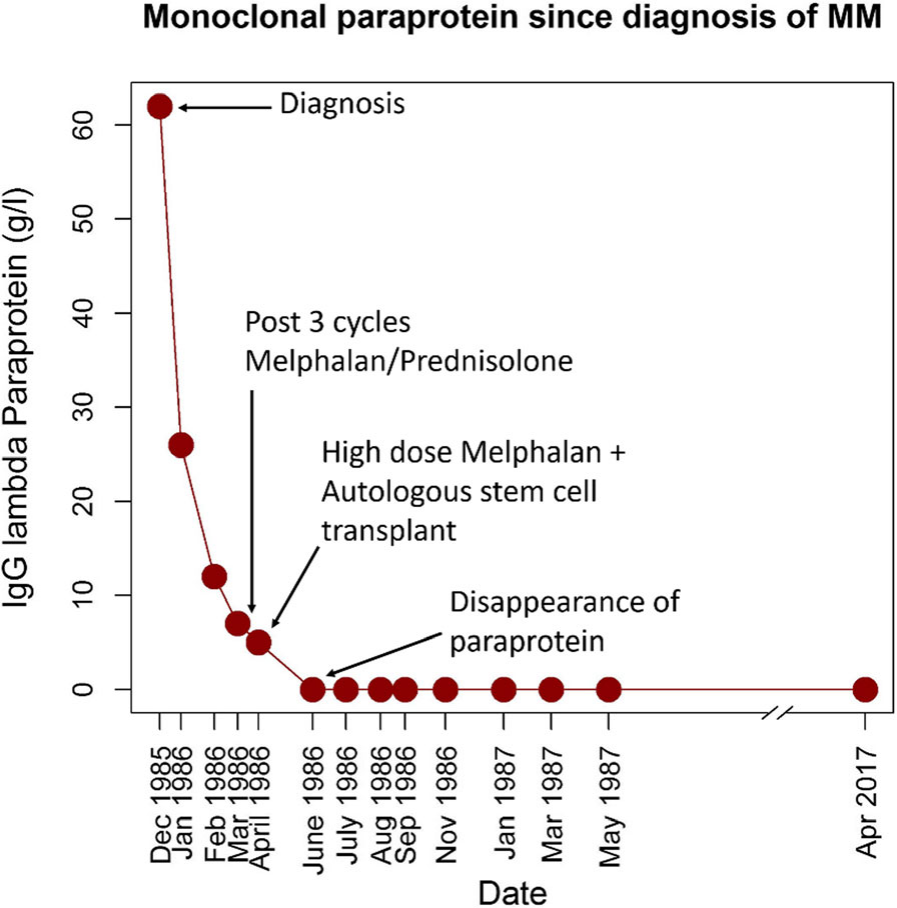
Level of IgG lambda paraprotein (g/l) from diagnosis of myeloma

Since the patient met the established criteria for symptomatic MM [6] with end organ involvement as demonstrated by her anaemia, bony lytic lesions and immunosuppression with recurrent infections, she was commenced on standard chemotherapy advocated at the time. This comprised three cycles of melphalan 10 mg with prednisolone 60 mg for 4 days orally. After 1 cycle of chemotherapy the patient’s paraprotein had fallen to 26 g/l. She subsequently received two further cycles of melphalan and prednisolone which led to a further reduction in her paraprotein level to 7 g/l. This was followed by a high-dose melphalan (140 mg/m^2^) and autologous stem-cell transplant. Three months after her bone marrow transplant the patient’s paraprotein was undetectable and has never been detected again **(**Fig. 1 **)**.

The patient had been annually reviewed since diagnosis and has remained in complete remission 30 years later with no further chemotherapy for her MM. Specifically, in April 2017, she continued to have a normal haematological profile with a haemoglobin of 135 g/l, white cell count of 9.1 × 10^9^/l, and a platelet count of 191 × 10^9^/l)). She is no longer immunosuppressed with an IgA of 0.6 g/l and IgM of 0.5 g/l. She also has no detectable paraprotein with a normal light chain ratio as assessed by serum protein electrophoresis and serum free light chain assay last assessed in April 2017. A repeat bone marrow biopsy 25 years ago showed that the patient was in complete remission. Imaging by whole body MRI in 2016 revealed no evidence of MM.

**Fig. 2:**
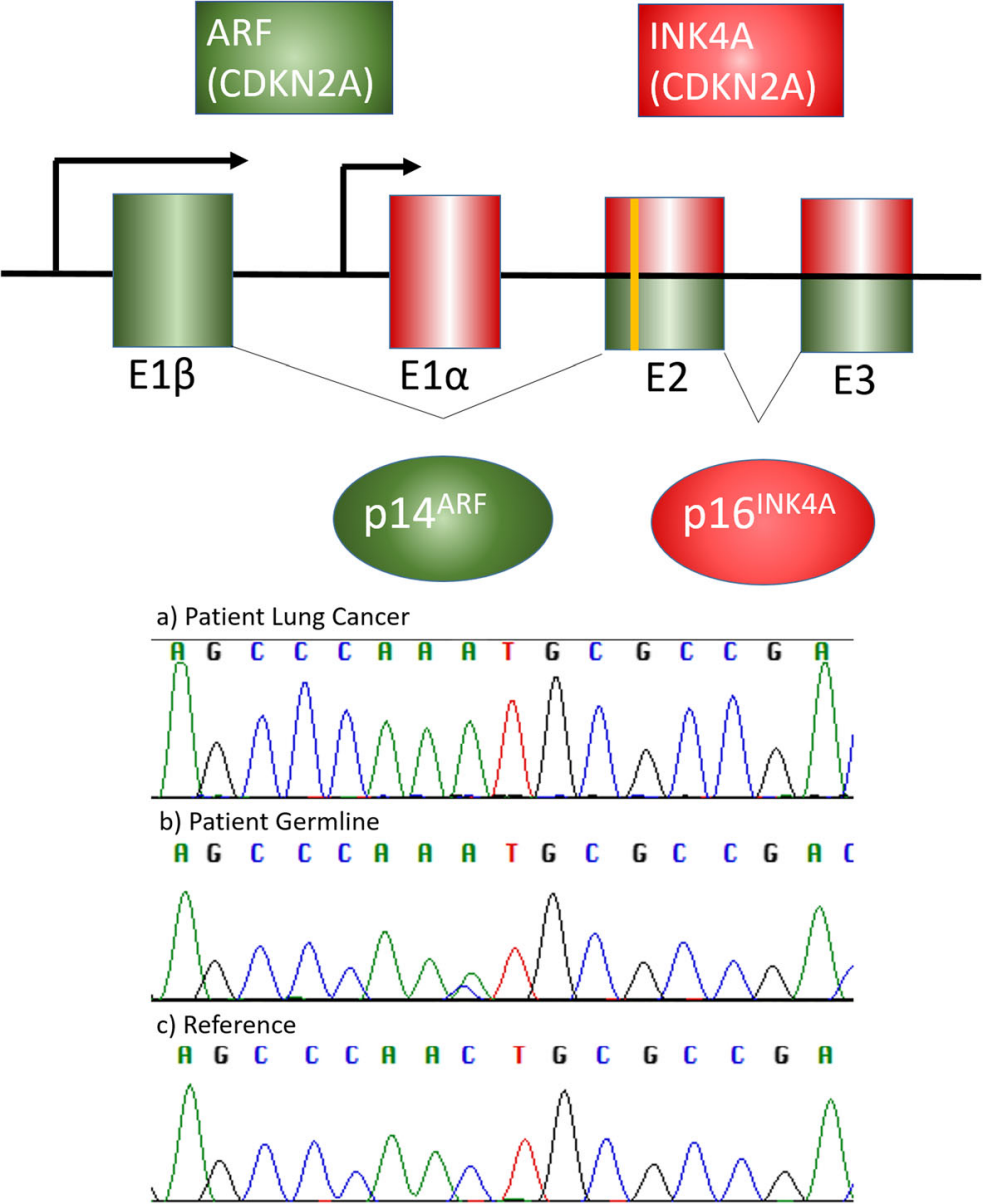
Chromatogram from Sanger sequencing showing pathogenic heterozygous c.213C > A mutation in *CDKN2A* of patient germline DNA, the homozygous A allele at c.213 representing loss of heterozygosity in the patient’s lung cancer tissue compared to reference sequence with diagrammatic representation of alternatively spliced products. The *CDKN2A* gene encodes both p14^ARF^ (green exons) and p16^INK4A^ (red exons), generating two transcripts that are translated in alternative reading frames

**Fig. 3:**
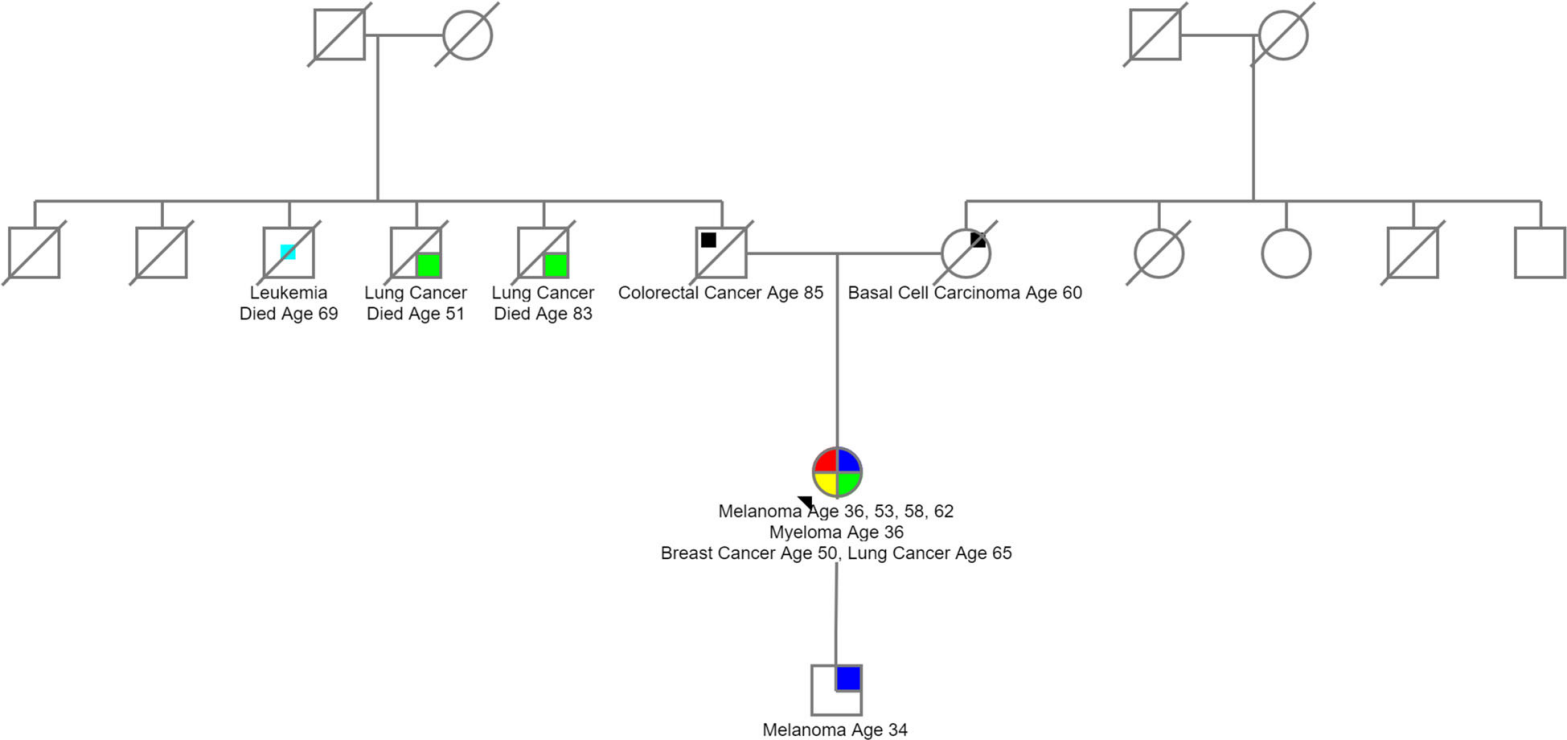
Patient pedigree

Five months prior to being diagnosed with MM she had been diagnosed with a superficial spreading malignant melanoma on her right leg, which was successfully treated by wide excision. She was subsequently diagnosed with three further primary melanomas at ages 53 (right buttock), 58 (right flank) and 62 (right forearm), all also successfully treated by surgical excision. While there was at that juncture no family history of melanomas or early onset pancreatic cancer, a diagnosis of hereditary melanoma syndrome which can be caused by germline mutations in the cyclin-dependent kinase Inhibitor 2A (*CDKN2A*) gene was considered in view of the history of multiple melanomas. Genetic testing of constitutional DNA extracted from EDTA venous blood was performed by genomic DNA PCR amplification using primers described previously of the 4 exons of CDKN2A (exons 1α, 1β, 2 and 3) [7]. PCR fragments were isolated by agarose gel electrophoresis and purified prior to Sanger sequencing using QIAquick Gel Extraction Kit (Qiagen, Paisley, UK). This revealed the patient was a heterozygous carrier of the pathogenic c.213C > A mutation in the *CDKN2A* gene. This mutation results in a missense substitution of the amino acid asparagine to lysine in the expressed INK4A protein at position 71(N71K) and a leucine to methionine substitution in the expressed ARF protein (L86M) **(**Fig. 2 **)**. The patient’s son has since been diagnosed with melanoma at the age of 34 years but he has yet to be genetically tested **(**Fig. 3 **)**. Otherwise the patient’s family history is unremarkable and specifically there is no evidence for propensity to pancreatic cancers in family members.

**Fig. 4:**
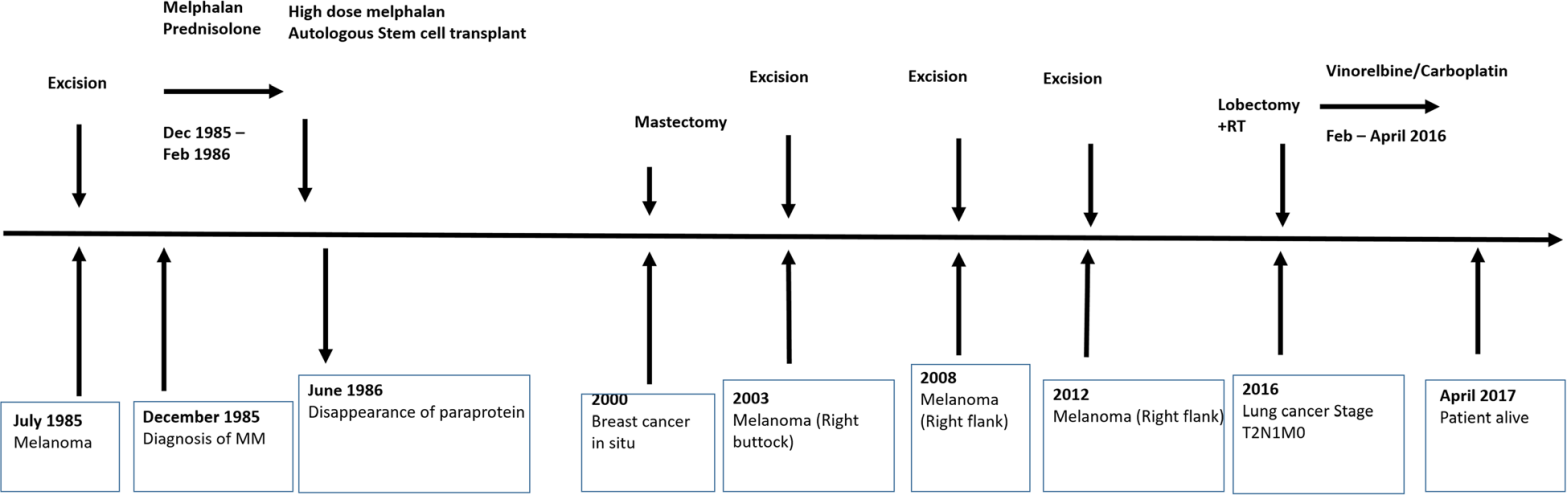
Timeline of primary malignancies and therapy

Aside from MM and melanoma the patient has also been diagnosed with two other cancers. Firstly, in situ breast cancer at the age of 50 incidentally discovered during routine breast screening and which was treated with a mastectomy. Secondly, stage T2bN1M0 adenocarcinoma of the lung at the age 66 which was diagnosed following whole-body diffusion-weighted MRI investigation, performed as part of her MM follow-up investigation of hip pain. Her lung carcinoma has been treated by lobectomy, adjuvant chemotherapy with carboplatin and vinorelbine in addition to radiotherapy **(**Fig. 4 **)**. Mutation testing of the patient’s lung cancer tissue by PCR amplification and Sanger sequencing as described above revealed a loss of heterozygosity of the C.213C > A allele compared to the patient’s germline DNA **(**Fig. 2 **)**. Paradoxically her MRI did not show any signs indicative of active MM.

## Discussion and conclusions

The pathogenic nature of the specific c.213C > A mutation in *CDKN2A* noted in this patient is suggested by the fact that it has been described previously in several hereditary melanoma families [8–10] as well as a supraglottic squamous cell carcinoma [11]. In silico predictions with the algorithms used by Polyphen-2, SIFT and mutation taster all indicate that this is a pathogenic mutation. Additionally, functional assays of the protein INK4A with this mutation also suggest pathogenicity [12]. The loss of the wild type allele in the patient’s lung cancer DNA as shown in Fig. 2 also suggests that this is a pathogenic mutation causing an increased susceptibility to tumours.

To our knowledge this is only the second case of a germline mutation in *CDKN2A* being reported in association with MM. The previous report described a MM patient who had a strong family history of melanoma consistent with a diagnosis of hereditary Melanoma Syndrome caused by a pathogenic exon 1 *CDKN2A* mutation. Loss of the wild-type allele was detected in malignant plasma cells consistent with *CDKN2A* acting as a tumour suppressor in the context of MM in this case report [13].

Typically, germline mutation of *CDKN2A* is associated with a restricted spectrum of cancers; primarily melanoma and pancreatic carcinoma. However, an increased risk of other cancers including childhood ones, lung, oropharyngeal and breast have been reported albeit at lower frequency [14]. Evidence for the association of the *CDKN2A* gene and its association with myeloma susceptibility has been shown in genome wide association studies which found a susceptibility locus for myeloma at chromosome 9p21.3 variant rs2811710 of *CDKN2A* [15]. A population based study in 1354 people with multiple myeloma also suggests a link between multiple myeloma, melanoma within first and second degree relatives [16]. This has been further confirmed in other studies [17–19].

Such data implies a wider impact of *CDKN2A* in tumour aetiology and although rare suggests the relationship with MM is not coincidental. It is perhaps not surprising that *CDKN2A* impacts on the aetiology of a wide range of tumour types. One of the gene transcripts *ARF* functions as a stabiliser of p53 through interaction with E3 ubiquitin protein ligase MDM2, thereby enhancing p53-dependent transactivation and apoptosis. Mutations in ARF result in destabilisation of p53. Abnormalities of p53 are present in almost all cancers. This can be direct via deletion/mutation or hypermethylation of the p53 promoter, altering its stabilisation through alterations/deletions of ARF or overexpression of MDM2 [20, 21] or via other mechanisms.

Alternate splice variant of *CDKN2A*, *INK4A* functions is a member of the cyclin dependent kinase inhibitors. It binds to CDK4 and CDK6 kinases and sequesters them from their regulatory cyclin D subunits. As a result, CDK4 cannot phosphorylate the retinoblastoma protein (Rb) which is considered the gatekeeper of cell proliferation As a result of mutations in INK4A, there is resultant dysregulation of cell cycle control and tumour proliferation [22].

Further evidence of the role of *CDKN2A* in tumour development comes from mice lacking ARF and/or INK4A which develop tumours early in life succumbing to lymphomas and fibrosarcomas [23]. Additionally, families with germline mutations of *CDKN2A* show increased rates of melanoma and pancreatic cancer but also have increased rates of other malignancies such as cancers of the breast, nervous system, GI tract, lymphoma and cervical cancers also suggesting that the increased susceptibility to cancer is not restricted to melanoma and pancreatic cancer alone [24, 25]. Furthermore, frequent somatic mutations and deletions of *CDKN2A* have been noted in several cancers including pancreatic adenocarcinoma, oesophageal and gastric carcinomas, leukemias and melanomas indicating its role in cancer pathogenesis [26, 27]. Deletions as well as mutations of the *Rb* as well as *TP53* are frequent in myeloma indicating a critical role of these genes in its pathogenesis [28]. It is therefore feasible that genes altering the function of these proteins will also increase the susceptibility to myeloma.

The case we report is striking in that after only melphalan therapy the patient has had a remission from MM of over 30 years and in essence is cured. Although speculative the observation is consistent with the patient’s MM being especially sensitive to alkylating chemotherapy. Melphalan causes DNA damage and subsequent cell death due to impairment in the DNA repair pathway. Studies have shown that mutations in *CDKN2A* increase sensitivity to chemotherapy [29, 30]. Moreover, MDM2 inhibitors increase sensitivity to conventional chemotherapy in different cancers. MDM2 targets p53 protein for proteosomal degradation and its function is inhibited by ARF. Mutations in ARF have been shown to destabilise p53 through this mechanism and the increased susceptibility to chemotherapy induced by MDM2 inhibitors may reflect why this patient also had a high sensitivity to conventional chemotherapy which causes DNA damage. Effectiveness of MDM2 inhibitors has also been demonstrated in haematological malignancies such as MM, AML and ALL. [31–33]. Of note in this regard is that AML with the translocation of t(8;21) resulting in the RUNX1-ETO fusion gene which directly inhibits ARF is one of the few subtypes of AML which can be cured by high dose chemotherapy alone [34].

In conclusion, outcome of high-dose chemotherapy in the patient we report has resulted in cure suggesting that such germline mutations may confer increased MM sensitivity to chemotherapy. This finding raises the possibility that long-term survivorship from MM in other patients may be the consequence of carrier status for tumour suppressor genes with biological relevance to DNA damage.

## Abbreviations

ALL: acute lymphoblastic leukemia
AML: acute myeloid leukemia
*CDKN2A*: cyclin-dependent kinase Inhibitor 2A
DNA: deoxyribonuelcic acid
EDTA: Ethylanediaminetatraacetic acid
*MDM2*: mouse double minute 2 homolog/E3 ubiquitin-protein ligase MDM2
MM: Multiple Myeloma
p53: tumour protein p53
RUNX1-ETO: Runt-related transcription factor 1-Eight Twenty-One oncoprotein
